# Assessing the relationship between maternal risk for attention deficit hyperactivity disorder and functional connectivity in their biological toddlers

**DOI:** 10.1192/j.eurpsy.2022.2325

**Published:** 2022-10-13

**Authors:** Anastasia Kerr-German, Stuart F. White, Hendrik Santosa, Aaron T. Buss, Gaelle E. Doucet

**Affiliations:** 1 Boys Town National Research Hospital, Center for Childhood Deafness, Language and Learning, Omaha, Nebraska 68131, USA; 2 Boys Town National Research Hospital, Institute for Human Neuroscience, Boys Town, Nebraska 68010, USA; 3 Department of Pharmacology and Neuroscience, Creighton School of Medicine, Omaha, Nebraska 68124, USA; 4 Department of Radiology, University of Pittsburg, Pittsburg, Pennsylvania 15260, USA; 5 Department of Psychology, University of Tennessee, Knoxville, Tennessee 37996, USA

**Keywords:** ADHD, attention, fNIRS, functional connectivity, passive viewing, toddlers

## Abstract

**Background:**

Attention deficit hyperactivity disorder (ADHD) is a neurodevelopmental disorder associated with increased risk for poor educational attainment and compromised social integration. Currently, clinical diagnosis rarely occurs before school-age, despite behavioral signs of ADHD in very early childhood. There is no known brain biomarker for ADHD risk in children ages 2–3 years-old.

**Methods:**

The current study aimed to investigate the functional connectivity (FC) associated with ADHD risk in 70 children aged 2.5 and 3.5 years via functional near-infrared spectroscopy (fNIRS) in bilateral frontal and parietal cortices; regions involved in attentional and goal-directed cognition. Children were instructed to passively watch videos for approximately 5 min. Risk for ADHD in each child was assessed via maternal symptoms of ADHD, and brain data was evaluated for FC.

**Results:**

Higher risk for maternal ADHD was associated with lower FC in a left-sided parieto-frontal network. Further, the interaction between sex and risk for ADHD was significant, where FC reduction in a widespread bilateral parieto-frontal network was associated with higher risk in male, but not female, participants.

**Conclusions:**

These findings suggest functional organization differences in the parietal–frontal network in toddlers at risk for ADHD; potentially advancing the understanding of the neural mechanisms underlying the development of ADHD.

## Introduction

Attention deficit hyperactivity disorder (ADHD) is a common disorder affecting 7–11% of US children [1, 2] and affects attentional abilities [3]. The diagnosis of ADHD typically occurs once children are in school and experience disruption to their academic and social lives [4, 5]. However, recent work looking at DSM-5 symptoms of ADHD in conjunction with temperament has demonstrated that children with ADHD show early behavioral signs of developmental delay during the preschool years [6, 7]. Such children often have parents with the disorder. For example, work linking genetics, heritability, and familial risk of ADHD has found that parents with ADHD are 50–80% more likely than parents without ADHD to have one or more children with the diagnosis [8–11]. Accordingly, neurodevelopmental disorders such as ADHD cluster in families due to shared environment and genes.

A wide variety of treatments for ADHD exist, though the long-term efficacy of these treatments is limited. 40–50% of individuals diagnosed with ADHD in childhood continue to meet criteria for ADHD and require treatment in early adulthood [12, 13]. Moreover, 20% of adults with childhood ADHD still show dysfunction even if they no longer meet criteria for ADHD [14, 15]. Long-term impairment in childhood ADHD continues into adulthood along domains of psychosocial, educational, and neuropsychological functioning regardless of medication or therapy interventions [16]. Further, longitudinal work looking at adult outcomes of childhood ADHD have concluded that family, behavioral, and neuropsychological factors not only contribute to later quality of life and success outcomes (i.e., occupational, education attainment, executive functioning) but are areas of research that will be critical for efficacious ADHD interventions in the future [17]. It is possible that treatment outcomes could be improved with a better understanding of the neural underpinnings of ADHD. A better understanding of dysfunctional neural systems may allow for improved and earlier identification of ADHD by providing biomarkers. Moreover, the potential for biologically informed subtyping based on neural-level individual differences may allow for the development of more individualized treatments.

One potential avenue for furthering our understanding of ADHD’s ontogeny is to examine resting-state functional connectivity (FC). FC is the temporal correlation of spatially distant neurophysiological events (e.g., [18]). A number of studies have found differences in FC within the attentional network (e.g., parietal cortex, lateral and medial frontal cortex) between controls and patients with ADHD in both children [19, 20] and adults [21]. Importantly, weaker FC between these specific regions has been associated with core aspects of ADHD symptomatology (i.e., poor attention, social functioning difficulty, and impaired cognitive control) in children and adolescents [22]. Assessing FC across the attentional network in very young children could be one avenue for comprehending the development of ADHD symptoms. Specifically, FC during a resting-state condition is much easier to measure in younger children compared to task-based activation (i.e., more potential for exclusion).

Further, there are notable sex differences in ADHD with respect to prevalence rates [23, 24] and treatment outcomes [25]. Such differences at the behavioral level may result from sex differences in brain activity in response to ADHD risk; however, neuroimaging studies remain inconsistent in this regard (e.g., [26] and [27]). Furthermore, due to the lack of neuroimaging data on preschool-aged children, it remains unknown whether sex differences emerge, if at all, in children who may develop ADHD, or if these differences are present in very young children regardless of ADHD risk.

Because of methodological constraints, little is known of the neural ontogeny of ADHD. Common task-based neuroimaging methods used on adults (e.g., magnetic resonance imaging (MRI)) are often challenging for children under 5 years old, primarily due to very young children moving too much to yield good data [28, 29–31]. Functional near-infrared spectroscopy (fNIRS) is a superior neuroimaging technique in very young children, because it is less influenced by head motion artifacts and is designed for a successful use in young children [32, 33].

In this context, the current study aimed to investigate the neural profile associated with risk factors for developing ADHD at the age of 2–3 years-old. fNIRS was used to assess FC between lateral frontal and parietal cortices during passive viewing of video clips. Risk for ADHD was determined via maternal adult symptoms of ADHD, because previous research has shown that maternal ADHD is more predictive of childhood ADHD symptomology than paternal ADHD [34]. We hypothesized that increased ADHD risk would be associated with weaker FC between the frontal and parietal cortices, which are typically associated with attentional control. In addition, we hypothesized that females, in comparison to males, will show stronger connections in these regions associated with increased risk for ADHD [27]. Finally, we hypothesized that a risk factor for ADHD would be associated with a temperament profile. Following previous work [35, 36], we predicted that ADHD-risk would be associated with lower scores on the composite score of effortful control and on the sub-scores of impulsivity, inhibitory control, frustration, and attentional focus.

## Methods

### Participants

Seventy children aged 2.5-years-old (*N* = 37, female = 21) and 3.5-years-old (*N* = 33, female = 15) were recruited. All participants’ age fell within ±6 weeks of the target ages of 30 (2.5 years) or 42 (3.5 years) months. The current sample size is justifiable based on previous work with functional connectivity, fNIRS, and very young children (e.g., [37]). All children included in the study had normal hearing and no known cognitive or neural developmental delays or abnormalities. Parental consent was obtained prior to the study. Throughout the procedures, continuous verbal assent was maintained with all children. The Institutional Review Board for Research with Human Subjects at the University of Tennessee, Knoxville, approved this study.

### Adult ADHD Self-Report Scale (ASRS-v1.1) Symptom Checklist

The ADHD self-report screening scale (ASRS v1.1) [38, 39] was given to the biological mother. This scale has been reliably associated with ADHD diagnoses in both adults and adolescents [38, 40, 41], and has been validated for use with both general and clinical populations [42]. Parents indicated their personal experience with symptoms of ADHD based on DSM-IV criteria. The mothers’ total score from the ASRS served as a metric of their child’s risk for ADHD, where 0 was no risk and 18 was the highest risk. Scoring was calculated as a frequency of items that met clinical criteria [38], and were further converted to *z* scores. Independent samples *t* tests on ADHD risk *z* scores were conducted between age bins (i.e., 2, 3).

### Temperament Scores

Parents also completed a short temperament questionnaire specifically designed for children aged 1–3 years old (Early Childhood Behavior Questionnaire [ECBQ] – Short Form [43]). Temperament scores were derived from the survey via standard scoring. The Childhood Behavior Questionnaire, a measure of temperament in children 3–7 years old, has been used in the past to gauge risk and predict likelihood of developmental psychopathology in young children [35, 36].

### Apparatus and Materials

**Figure 1.**
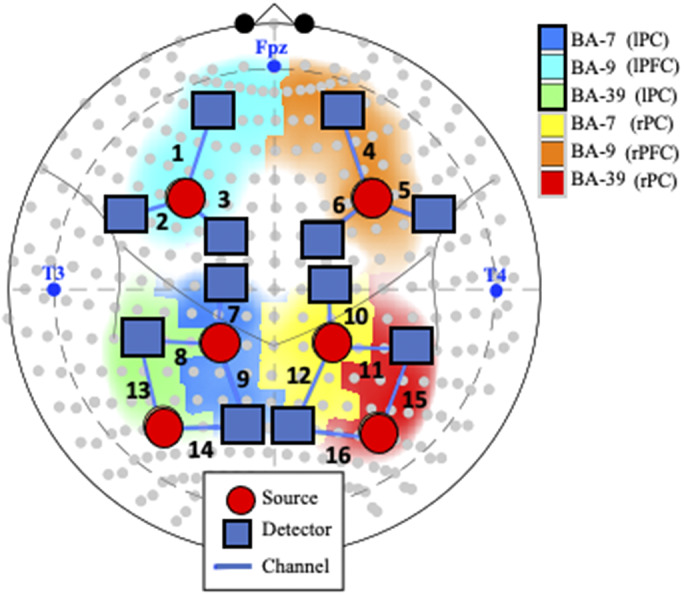
Probe and channel abbreviations. *Note.* fNIRS probe placement in the International 10–10 System, represented by gray dots in the current schematic. Blue text represents landmarks (i.e., nasion [Fpz], left preauricular [T3], and right preauricular [T4]). Positioning of the 18 optodes, consisting of 6 sources (orange circles) and 12 detectors (blue squares), creates 16 channels (numbered in black text). *Abbreviations:* BA: Broadman Area; L: Left; R: Right; lPF: left parietal cortex; rPC: right parietal cortex; lFC: left frontal cortex; rFC: right frontal cortex.

The experimental data were recorded using a continuous NIRS imaging system (TechEn Inc., Milford, MA) at a sampling rate of 25 Hz. The data were measured simultaneously at two wavelengths (690 nm and 830 nm). The system automatically adjusted light intensity to provide optimal gain. The optodes were placed over the dorsolateral prefrontal and parietal cortex (Figure 1). The distance between source and detector was 30 mm. This probe was scaled for both a 52 cm (*N* = 23) and 54 cm (*N* = 47) hat to account for head size differences across these two age groups. Among the children that used the 52 cm hat, 12 were 2.5 year old and 11 were 3.5 year old. Participants were placed between 63.5 and 65 cm from a computer monitor. Videos played on a 530 mm × 330 mm monitor with a resolution of 1280 × 960 pixels.

### Procedure

A session required two trained researchers: one to administer the passive watching paradigm and one to run the fNIRS machine. A blackout curtain was then positioned to cover half of the opening between the parental seating area and the child testing area to reduce artifact due to lighting. One researcher was stationed behind an occluder, where the fNIRS acquisition computer and machine were located behind the child. The other researcher sat off to the right side of the child, outside of their peripheral vision, to redirect them to the screen if they looked away and to provide a whispered reminder to “be quiet and calm” and “watch the video”.

### Passive Watching Paradigm

**Figure 2.**
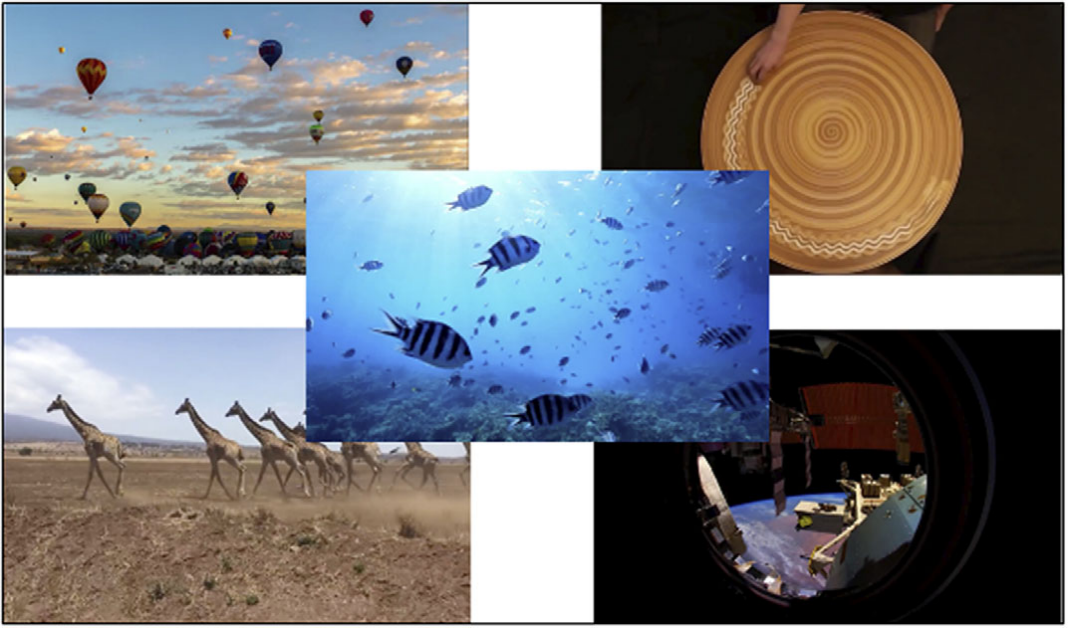
Still shots taken from the five passive-viewing paradigm videos.

We used a passive watching paradigm because it has been suggested that passively viewing a video is roughly analogous to rest in young children [44]. In the passive video-watching task, participants were given the following instructions: “You will be watching some movies. While you are watching, I want you to be still and calm, so you will need to be very still and calm before I start the movies. Remember to keep your eyes on the screen while the movies are playing.” Three to five soothing videos were randomly chosen, each with instrumental music that was temporally synchronized with the movement within each video. The volume of auditory components of the video was stationary and set to be clearly audible over the noise produced by the fNIRS machine (68–70 dB) from where the participants sat. Each video lasted 45–90 s (Figure 2).

No child received all video clips. A white central fixation was presented on a black screen between videos, and recordings from this time were not included in the concatenated data analyses. This task resulted in an accumulated recording of 4 min for each participant while viewing sessions lasting between 270 and 450 s. Given that an intertrial central fixation was used to reorient children to the screen and transition to the next video segment, some individual variability in the overall recording session duration did occur. While using this passive viewing paradigm, it is important to note that collecting resting-state measures in very young children has been challenging (e.g., [45]). Care was taken to extensively pilot the video stimuli used in this study to ensure moderate interest from children without inducing excitement or over-engagement. Stimuli were chosen based on soothability and potential to synchronize with classical music. All music selections had slow tempo and were synchronized with motion in the video to avoid violation of expectations or brain activity related to perceptual asynchrony (e.g., [46]).

### fNIRS Data Preprocessing

We obtained at least 30 s of recording prior to the first video, and only data from the passive viewing task were included in further analyses. Raw fNIRS signals were first resampled to 4 Hz and converted to changes in optical density. The measured intensity data of the two wavelengths were then converted to relative HbO_2_ (i.e., oxygenated hemoglobin) and HbR (i.e., deoxygenated hemoglobin) concentration changes using the modified Beer–Lambert law [47]. In order to extract the individual FC measures, we took a robust correlation approach and implemented the iterative autoregressive least-square technique (see associated literature for more details [48]). A false discovery rate (FDR) correction was further applied by calculating the robust correlation coefficient of the temporally whitened signals. No participants were excluded from analyses (i.e., inclusion criteria after pre-processing of SNR ≥80% on at least 11/16 channels). A study by Santosa et al. [48] showed the robustness of this algorithm and demonstrated that it yields more reliable estimates to serially correlated errors and statistical outliers due to motion artifacts compared to other approaches (i.e., “Temporal Derivative Distribution Repair” [49]).

### Statistical Analyses

At the behavioral level, we investigated the association between the composite and subscale sub scores from the ECBQ and the ADHD risk scores by conducting partial Pearson correlation analyses where age was controlled for. Two sample *t* tests were conducted on all subscales and composite scores to test for differences between the two age groups.

Using the fNIRS data, we tested the effects of ADHD risk, sex, and their interaction on FC, with age added as a covariate, by conducting analyses of covariance. The two age groups were combined in all analyses because they did not differ in ADHD risk or sex. A FDR correction (i.e., function FDDR in AnalyzIR toolbox) was further applied to correct to reduce false discovery rate in FC (e.g., [37]). We only considered HbO_2_-to-HbO_2_ correlations, because there is little variability in HbR and there is a continuing debate on whether HbR is informative in FC analyses [50]. All first and second level statistical analyses were implemented in MATLAB™ version 2020a as part of an open-source AnalyzIR toolbox [51].

## Results

### Behavioral Analyses

The two groups of children of 2.5 and 3.5 years of age did not significantly differ in their ADHD risk scores [*t* (66) = −.214, *p* = .831] nor frequency of sex [*X* ^2^ = .672, *p* = .413]. Information about the number of parents in the household (single or dual), highest level of parental education, household income per year, number of siblings in the home, and childcare currently being utilized for the participant during the day is provided in Table 1.

**Table 1.** Family Information in each study sample.

		2.5-years-old (n = 37)		3.5-years-old (n = 33)	
		Male	Female	Male	Female
Household Size	Single Parent	1	3	1	1
	Dual Parent	15	18	16	14
Highest Parent Education	High School or equivalent	2	2	0	2
	Associates or Technical Training	2	5	2	2
	Bachelors	3	6	5	2
	Master’s Degree	4	4	3	6
	Doctoral Degree	5	4	7	3
Household Income Per Year	< $18,650	1	0	0	0
	$18,650–$75,900	7	12	3	7
	$75,900–$153,000	4	5	10	5
	> $153,000	4	3	4	3

Based on standard scoring, eight mothers exceeding the clinical-cutoff score for ADHD, indicating a likely clinically meaningful range of risk scores. Of their biological children, four children were 2.5 years-old (female = 4) and four children were 3.5 years-old (female = 1). In addition, risk was not associated with any household/family demographics.

Next, we investigated the relationship between risk and temperament, in the whole sample. Two of the 15 subscales from the ECBQ were statistically different between age bins at an uncorrected level [fear, *t* (66) = 2.23, *p* = .029; and inhibitory control, *t*(66) = −2.02, *p* = .048]. Parents reported less fear and greater inhibitory control in older children compared to younger children. However, these differences were not retained after applying a Bonferroni correction for multiple comparisons (Table 2) [26]. ADHD risk level correlated with several temperament subscales: inhibitory control, *R* = −.328, *p* = .006; frustration, *R* = .288, *p* = .016; activity level, *R* = .400, *p* = .001; high intensity pleasure, *R* = .292, *p* = .014; perceptual sensitivity, *R* = .276, *p* = .021; sadness, *R* = .248, *p* = .039; and surgency, *R* = .298, *p* = .012; Bonferroni corrected *p*-values.

**Table 2.** Descriptive statistics for each age sample.

Demographics	2.5 years old		3.5 years old		
N	37		33		
Female (%)	57.80%		46.90%		
Survey Data	*M*	*sd*	*M*	*sd*	*p* *
*ASRS V1.1*					
Maternal ADHD Risk	3.757	3.338	3.750	3.756	0.831
*ECBQ*					
Activity Level	4.863	0.821	4.766	0.878	0.486
Attentional Focus	4.629	0.907	5.041	0.701	0.065
Attentional Selectivity	4.714	0.765	4.921	0.824	0.187
Cuddliness	5.087	0.864	5.478	0.913	0.168
Discomfort	2.947	1.251	2.634	1.104	0.338
Fear	2.999	1.141	2.472	0.837	0.029*
Frustration	4.222	1.211	3.747	1.121	0.077
High Intensity Pleasure	5.469	0.845	5.264	0.934	0.262
Impulsivity	3.943	1.112	3.917	0.966	0.905
Inhibitory Control	3.924	1.027	4.325	0.843	0.048*
Low Intensity Pleasure	4.999	0.732	5.039	0.946	0.990
Motor Activation	2.848	1.102	2.598	0.985	0.506
Perceptual Sensitivity	4.692	0.848	4.400	0.876	0.129
Positive Anticipation	5.777	1.022	6.109	0.693	0.175
Sadness	3.296	0.778	3.092	0.865	0.570
Shyness	3.678	1.195	3.459	1.091	0.241
Sociability	5.653	1.073	5.598	0.820	0.791
Soothability	4.587	1.070	4.503	0.948	0.761
Negative Affect	3.526	0.716	3.237	0.623	0.082
Surgency	5.146	0.604	5.131	0.526	0.761
Effortful Control	4.684	0.638	4.960	0.632	0.089

* Uncorrected *p*-values, *p < 0.05

### ADHD Risk and Sex Effects on Functional Connectivity

**Figure 3.**
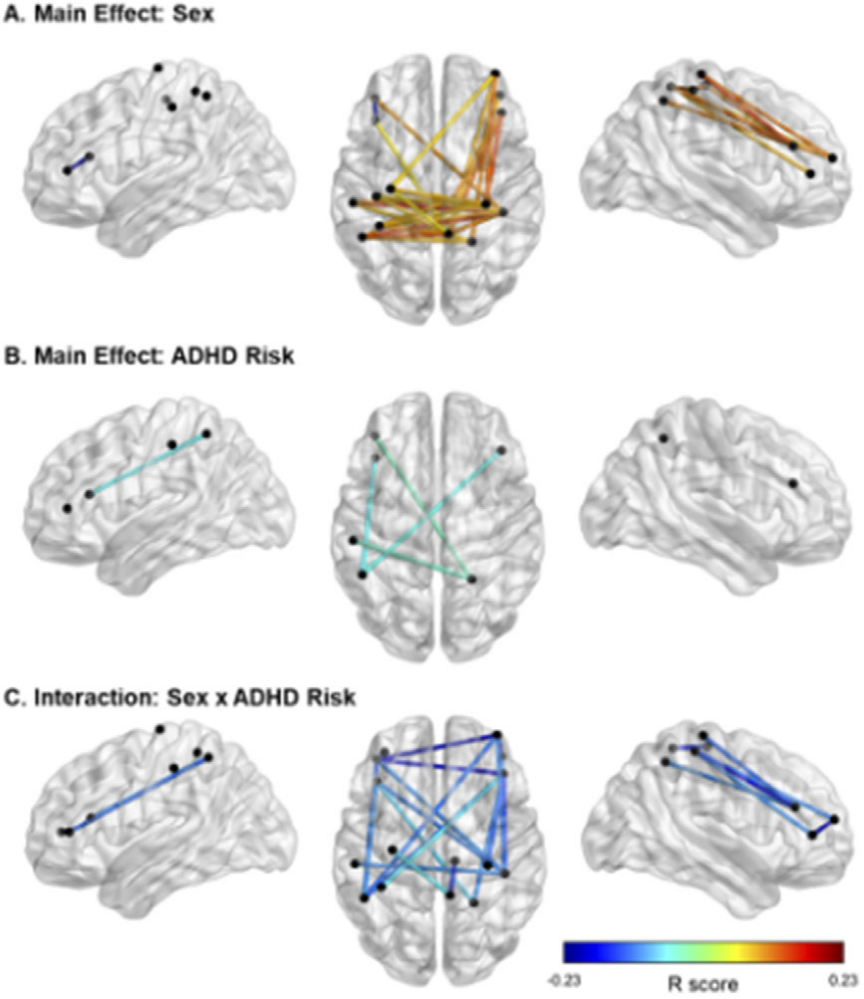
Significant effects of ADHD risk and sex on FC. *Note.* In panel A, cooler colors reflect higher FC in females than in males, and warmer colors reflect higher FC in males than females. In panels B and C, cooler colors indicate negative *R* values and therefore lower FC in relation to higher risk for ADHD. In Panel C, all FC links displayed are in males only. Location of the nodes is illustrative only, based on approximate MNI coordinates for the current probe (i.e., channels 1–16; see Figure 1). The midpoint location between each source and detector was calculated in AtlasViewerGUI within the Homer3 software package in MATLAB. BrainNet Viewer was used to visualize significant FC.

fNIRS results showed significant main effects of ADHD risk and sex as well as a significant interaction between these two factors (Figure 3, Tables 3–5).

**Table 3.** Main Effect of Sex on FC: Differences in FC between males and females.

Channel Pair		Regions	R	*t*	p*
2	3	lPFC<−>lPFC	−0.222	−2.062	0.044
2	15	lPFC<−>rPC	0.109	2.025	0.048
3	12	lPFC<−>lPC	0.068	2.074	0.043
4	7	rPFC<−>rPFC	0.075	2.155	0.036
4	11	rPFC<−>rPFC	0.117	2.020	0.049
4	12	rPFC<−>lPC	0.093	2.090	0.042
4	16	rPFC<−>rPC	0.110	2.861	0.006
5	12	rPFC<−>lPC	0.088	2.014	0.049
6	10	rPFC<−>lPC	0.113	2.124	0.038
6	11	rPFC<−>rPC	0.143	2.501	0.016
6	12	rPFC<−>lPC	0.127	3.049	0.004
7	10	lPFC<−>lPFC	0.092	2.416	0.019
7	11	lPC<−>rPC	0.088	2.045	0.046
7	12	lPFC<−>lPFC	0.070	2.231	0.030
7	15	lPC<−>rPC	0.087	2.570	0.013
8	12	rPC<−>lPC	0.078	2.812	0.007
8	15	rPC<−>rPC	0.080	2.053	0.045
9	10	lPFC<−>lPFC	0.089	3.021	0.004
9	11	lPC<−>rPC	0.087	2.139	0.037
9	12	lPC<−>lPC	0.108	3.518	0.001
9	15	lPC<−>rPC	0.091	2.768	0.008
10	13	lPC<−>rPC	0.095	2.048	0.046
10	14	lPC<−>rPC	0.105	2.476	0.017
10	15	lPC<−>rPC	0.117	2.243	0.029
12	14	lPC<−>rPC	0.138	3.202	0.002
12	15	lPC<−>rPC	0.113	2.392	0.020
13	15	rPC<−>rPC	0.169	2.774	0.008
13	16	rPC<−>lPC	0.086	2.733	0.009
14	15	rPC<−>rPC	0.151	2.821	0.007
14	16	rPC<−>lPC	0.093	2.130	0.038

*Note.* Positive values represent: Males > Females while negative values represent: Females > Males. All analyses were controlled for age; *FDR corrected *p*-values.

*Abbreviations:* rPC = Right Parietal Cortex; lPC = Left Parietal Cortex; rPFC = Right Prefrontal Cortex; lPFC = Left Prefrontal Cortex.

**Table 4.** Main Effect of ADHD on FC.

Channel Pair		Regions	R	*t*	p*
2	16	lPFC<−>rPC	−0.031	−2.154	0.036
3	15	lPFC<−>rPC	−0.061	−2.537	0.014
6	15	rPFC<−>rPC	−0.054	−2.060	0.044
13	16	lPC<−>rPC	−0.039	−2.580	0.013

*Note.* All analyses were controlled for age; *FDR corrected *p*-values.

*Abbreviations:* rPC = Right Parietal Cortex; lPC = Left Parietal Cortex; rPFC = Right Prefrontal Cortex; lPFC = Left Prefrontal Cortex.

**Table 5.** Sex by ADHD Risk Interaction.

Channel Pair		Regions	R	*t*	p*
1	2	lPFC<−>lPFC	−0.204	−2.008	0.050
2	4	lPFC<−>rPFC	−0.206	−2.584	0.013
2	6	lPFC<−>rPFC	−0.190	−2.324	0.024
2	14	lPFC<−>rPC	−0.131	−2.585	0.013
2	15	lPFC<−>rPC	−0.109	−2.069	0.044
3	14	lPFC<−>rPC	−0.108	−2.442	0.018
3	15	lPFC<−>rPC	−0.118	−2.386	0.021
3	16	lPFC<−>rPC	−0.087	−2.360	0.022
4	5	rPFC<−>rPFC	−0.210	−2.035	0.047
4	14	rPFC<−>rPC	−0.126	−2.701	0.009
4	15	rPFC<−>rPC	−0.117	−2.070	0.043
5	15	rPFC<−>rPC	−0.112	−2.059	0.045
6	9	rPFC<−>lPC	−0.061	−2.258	0.028
6	11	rPFC<−>rPC	−0.134	−2.390	0.021
6	14	rPFC<−>rPC	−0.125	−2.947	0.005
6	15	rPFC<−>rPC	−0.166	−3.138	0.003
6	16	rPFC<−>rPC	−0.116	−2.861	0.006
7	12	lPC<−>rPC	−0.071	−2.303	0.025
10	12	lPC<−>lPC	−0.170	−2.151	0.036
12	10	lPC<−>lPC	−0.170	−2.151	0.036
14	15	lPC<−>rPC	−0.112	−2.133	0.038

*Note.* All effects are specific to the male participants. No significant effects were detected in females. All analyses were controlled for age; *FDR Corrected p-values.

*Abbreviations:* rPC = Right Parietal Cortex; lPC = Left Parietal Cortex; rPFC = Right Prefrontal Cortex; lPFC = Left Prefrontal Cortex.

With regards to the main effect of sex, females had one significant connection with higher FC within left frontal cortex, compared to males. In comparison to females, males showed higher FC in a widespread network largely covering the right frontal and bilateral parietal cortices (Figure 3A). Figure 3B depicts the main effect of ADHD risk on FC. Higher ADHD risk was associated with lower FC between (a) the left frontal cortex and both left and right parietal cortices, (b) the right frontal and right parietal cortices, and (c) the right and left parietal cortices (Table 4).

Lastly, Figure 3C depicts the interaction between sex and ADHD risk (Table 5). In detail, male participants showed decreased FC in a widespread bilateral network between the prefrontal and parietal cortices as ADHD risk increased. In contrast, no significant association between FC and ADHD risk was detected in female participants.

## Discussion

To our knowledge, the current study is the first to demonstrate that ADHD risk may be associated with changes in FC in children as young as 2.5 years old. Specifically, this is the first investigation of maternal ADHD symptom expression as it relates to differences in brain connectivity in their biological toddlers. Our results showed a main effect of ADHD risk with reduced FC between bilateral parietal and frontal cortices, with higher ADHD risk. Furthermore, a significant sex-by-ADHD risk interaction suggested that this was mainly driven by males. That is, the interaction of sex-by-ADHD risk demonstrated with higher risk for ADHD was associated with weaker connections between frontal and parietal cortices in males only. We also found an overall main effect of sex regardless of ADHD risk indicating that females had higher connectivity within the left frontal cortex compared to males, and that males showed widespread higher connectivity between right frontal and bilateral parietal cortices in comparison to females. This finding may indicate that males and females at higher risk for ADHD might be demonstrating different neural profiles in early life.

Consistent with our hypotheses, the current data indicate that increased ADHD risk was associated with reduced FC between frontal and parietal cortices, though this finding was restricted to males. This may seem contradictory with other studies that have reported such relationships in females (e.g., [52]). Rosch et al. [52] reported that girls, but not boys, with ADHD aged 8–12 showed atypical FC between the striatum and frontal regions, with it being related to delay discounting. However, another study done on children in the same age range reported the inverse with increased frontal-limbic striatum connectivity among boys with ADHD only [53]. Given fNIRS does not allow to collect information on subcortical regions, it is difficult to provide a clear explanation on the reasons of such discrepancies and whether they are based on different methodologies or whether higher ADHD risk impacts the brain in females at an older age. Future studies done on larger samples of young children should be conducted to test these discrepancies.

However, it is important to note that the sex-by-ADHD risk interaction findings reported in the current study are not inconsistent with previous studies (see [53]). A recent EEG study conducted by Kim et al. [54] reported that male patients with ADHD showed altered cortical network in the high beta band, which suggests that these attenuated network inefficiencies may lead to suboptimal information processing and affect symptoms of ADHD, such as inattention and hyperactivity. Other EEG studies pointed out that adult males with ADHD showed increased theta activity, but not females, indicating that distinct mechanisms may underpin adult ADHD in males and females [55, 56]. Together, this underscores the need for sex-specific investigation of ADHD, as patients may have distinct neural signatures, even at an early age.

While not a main question of the current study, the main effect of sex was also investigated given the report of sex differences in FC using resting-state functional MRI data in typically developing children [57]. In a sample of 7–18-year-old children, females showed more broadly distributed stronger connections in comparison to males, particularly in the left hemisphere [58], which is also what our data showed (Figure 3A). However, we also reported larger FC in males than females. This is in line with the study by Satterthwaite et al. [57] which reported sex differences within several brain networks, including the ventral attention network. Furthermore, they showed that males demonstrated more between-network connectivity than females. The sex differences observed in the current study and in these other studies may likely be a function of the developmental ranges assessed or the methodology, as fNIRS is unable to cover the whole brain and clearly identify distinct brain networks, especially at such a young age during which brain development is in full progress. Altogether, our findings support that sex differences in FC, regardless of ADHD risk status, are present at a very early age.

### Limitations

The current data should be considered in light of some limitations. First, our sample size is modest. However, given the importance of early development and the difficulty in obtaining neuroimaging data in very young children, even a modest sample is informative. Second, the current sample is predominantly homogenous along demographics dimensions (e.g., upper middle class Caucasian families, and dual family households), which potentially reduces the generalizability of the findings. Such limited variability reduces the likelihood that the current findings can be attributed to demographic variability; however, the role of demographic variables will need to be examined explicitly in future research.

Third, the current study did not comprehensively or directly assess the risk for ADHD and therefore, the current findings should be interpreted cautiously. Maternal ADHD symptoms were selected as a risk proxy as they have been previously used as a contributing factor to childhood ADHD symptomology [59]. Additionally, we examined temperament scales that have been associated with ADHD risk in other samples (e.g., [7]). Previous works have found that the effortful control composite score and the sub-scores of impulsivity, inhibitory control, frustration, and attentional focus were distinguishing temperamental factors in youth with ADHD [35, 36]. However, there are limitations to the use of temperament scales. While we found that, as predicted, inhibitory control and frustration were significantly associated with maternal risk as predicted, impulsivity, attentional focus, and effortful control were not associated with maternal ADHD symptoms. Moreover, activity level, high intensity pleasure, perceptual sensitivity, sadness, and surgency were also significantly associated with maternal ADHD symptoms. Future work might utilize additional and more comprehensive assessments of familial risk, such as a sample of clinically diagnosed mothers, or longitudinal data following children until an age where ADHD diagnosis is possible. Moreover, other psychopathology, such as depression and anxiety, was not assessed in either mothers or participants. Future work should directly assess psychopathology in the participants and gather substantially more data from parental and familial sources to assess risk for developing ADHD. These factors limit the degree to which we can be confident that the observed neural findings are specifically related to ADHD.

Last, the cross-sectional nature of the study is a limitation. Not all children in our risk group will go on to get a diagnosis of ADHD (e.g., [60, 61]). Determining whether the current findings are truly relevant to the risk of developing ADHD will require longitudinal work that explicitly tracks clinically relevant outcomes (e.g., developing ADHD). However, the current findings will be useful in guiding the development of this type of study.

## Conclusion

Overall, ADHD risk in the current study is associated with FC changes in male toddlers. The data suggest that male children, but not female children, as young as 2.5 years-old may show altered FC profiles in the attentional network in association with ADHD risk. These data suggest the use of fNIRS in very young children may be useful in providing information about early risk for psychopathology. Neuroimaging work in very young children can and should inform future work on developmental trajectories to psychiatric diagnoses, including ADHD, clinical assessment, and intervention.

## Data Availability

Due to the sensitive nature of the questions asked in this study, survey respondents were assured raw data would remain confidential and would not be shared with identifying information. Deidentified neural and survey data will be shared upon request from journal or for use in meta-analysis.
